# Prioritization of risk groups for influenza vaccination in resource limited settings – A case study from South Africa

**DOI:** 10.1016/j.vaccine.2018.11.048

**Published:** 2018-11-22

**Authors:** Meredith L. McMorrow, Stefano Tempia, Sibongile Walaza, Florette K. Treurnicht, Wayne Ramkrishna, Eduardo Azziz-Baumgartner, Shabir A. Madhi, Cheryl Cohen

**Affiliations:** a Influenza Division, Centers for Disease Control and Prevention, Atlanta, GA, United States; b Influenza Program, Centers for Disease Control and Prevention, Pretoria, South Africa; c U.S. Public Health Service, Rockville, MD, United States; d Centre for Respiratory Diseases and Meningitis, National Institute for Communicable Diseases of the National Health Laboratory Service, Johannesburg, South Africa; e School of Public Health, Faculty of Health Sciences, University of the Witwatersrand, Johannesburg, South Africa; f Communicable Disease Cluster, National Department of Health, South Africa; g Medical Research Council, Respiratory and Meningeal Pathogens Research Unit, University of the Witwatersrand, Johannesburg, South Africa; h Department of Science and Technology/National Research Foundation: Vaccine Preventable Diseases, University of the Witwatersrand, Johannesburg, South Africa

**Keywords:** Influenza, Vaccine policy, Hospitalization, Mortality, South Africa

## Abstract

**Background:**

Due to competing health priorities, low- and middle-income countries (LMIC) may need to prioritize between different influenza vaccine risk groups. Risk group prioritization may differ in LMIC based upon programmatic feasibility, country-specific prevalence of risk conditions and influenza-associated morbidity and mortality.

**Methods:**

In South Africa, we collected local disease burden data (both published and unpublished) and published vaccine efficacy data in risk groups and healthy adults. We used these data to aid policy makers with risk group prioritization for influenza vaccination. We used the following formula to assess potential vaccine averted disease in each risk group: rate of influenza-associated hospitalization (or death) per 100,000 population * influenza vaccine efficacy (VE). We further estimated the cost per hospital day averted and the cost per year of life saved by influenza vaccination.

**Results:**

Pregnant women, HIV-infected adults, and adults and children with tuberculosis disease had among the highest estimates of hospitalizations averted per 100,000 vaccinated and adults aged 65 years and older had the highest estimated deaths averted per 100,000 vaccinated. However, when assessing both the cost per hospital day averted (range: USD148–1,344) and the cost per year of life saved (range: USD112–1,230); adults and children with TB disease, HIV-infected adults and pregnant women had the lowest cost per outcome averted.

**Discussion:**

An assessment of the potential disease outcomes averted and associated costs may aid policymakers in risk group prioritization for influenza vaccination.

## 1. Introduction

In 2012, the World Health Organization (WHO) published a revised position paper on the use of influenza vaccines [1]. Collection of country-specific data on risk groups, influenza disease burden and vaccine cost-effectiveness was encouraged to aid in policy development, especially in low- and middle-income countries (LMIC). Risk groups to be considered for vaccination included pregnant women, children aged 6–59 months, adults aged 65 years and older, individuals with specific chronic medical conditions, and healthcare workers. Given available data, WHO’s Strategic Advisory Group of Experts on Immunization recommended the prioritization of pregnant women for seasonal influenza vaccination to protect both pregnant women and young infants against laboratory-confirmed influenza. As there were limited data available about influenza in pregnancy in LMIC, this recommendation was largely based on programmatic feasibility [2], the findings of a single randomized controlled trial from Bangladesh [3,4], and observational data on vaccine safety and effectiveness from high-income countries [5–7]. Influenza surveillance capacity in LMIC has expanded since the 2009 influenza A(H1N1) pandemic [8–10]. There is a growing body of literature about the burden of and risk groups for influenza-associated severe disease and death in LMIC [11–14]. In South Africa, as in high-income countries, young children and adults aged ≥65 years share a disproportionate burden of influenza-associated hospitalizations and deaths [15,16]; however, HIV-infection and tuberculosis (TB) disease are also important risk factors for severe outcomes in this context [17–20]. Thus, risk group prioritization may differ in LMIC based upon country-specific prevalence of risk conditions and influenza-associated morbidity and mortality.

Due to competing health priorities, LMIC may have limited resources to dedicate to the purchase of influenza vaccines. In such cases, vaccine needs of high risk groups may exceed the available resources for vaccine procurement. Prioritization among high risk groups may therefore be necessary to optimize influenza vaccine distribution in order to achieve the greatest impact on severe disease outcomes. In addition to assessing the burden of severe disease among different risk groups, the efficacy and effectiveness of influenza vaccines in the high risk group may also be relevant to risk group prioritization. There is evidence of reduced vaccine efficacy in children aged 6–23 months [21,22], adults aged 65 years and older [23] and individuals with specific chronic medical conditions (e.g. renal disease)[24]; however, influenza vaccine efficacy in pregnant women appears comparable to that of healthy adults and also prevents influenza in the young infant [15,25]. There are no data on the efficacy of influenza vaccines in persons with TB disease. Furthermore, risk groups with existing platforms for immunization service delivery (e.g. infants through the Expanded Programme on Immunisation (EPI) and pregnant women through antenatal care (ANC)) may be easier to access than groups that do not present for frequent, scheduled visits (e.g. the elderly). HIV-infected adults and persons with TB disease not receiving care are potentially at even higher risk of influenza-associated hospitalization or death than those in care [26,27] due to immunosuppression, but there is no existing mechanism to provide influenza vaccine to these high risk individuals. HIV and TB clinics may also be platforms for immunization service delivery; however, immunization services may not currently exist in these clinics. Likewise, persons aged 65 years and older are less likely to seek care [28]. There are limited existing data on influenza vaccine cost-effectiveness in LMIC [29]. Due to the lower cost of hospitalization and outpatient consultation, influenza vaccines may not be as cost effective in LMIC as in high-income countries. Likewise, high rates of unemployment may reduce influenza-associated productivity losses.

Given the complexities associated with influenza vaccine prioritization, we sought to aid policy makers to make evidence-based decisions on risk group prioritization in South Africa.

## 2. Methods

Using locally available disease burden data (both published and unpublished) and published literature on vaccine efficacy in high risk groups and healthy adults, we developed an evidence-based approach to aid policy makers in South Africa with risk group prioritization for influenza vaccination. For each risk group (pregnant women, HIV-infected adults, children aged 6–23 months^1^, adults aged 65 years and older, healthcare workers, adults and children with tuberculosis disease (all ages), and adults and children aged 5–64 years with non-HIV chronic illness) we sought to identify data on risk of hospitalization or death from influenza infection, population size, influenza vaccine efficacy (VE) and existing platform(s) for service delivery. VE estimates from randomized controlled trials were preferred over observational VE estimates. When no data were available (e.g. current influenza vaccine coverage) we used national estimates of influenza vaccine coverage from prior campaigns, or estimates of vaccine coverage from other vaccines targeting similar risk groups (e.g. tetanus vaccine coverage for pregnant women). We did not attempt to assess the potential impact of vaccination on disease transmission or to weigh the potential benefit of maternal influenza immunization on birth outcomes [30]. The data sources used in this study are provided in Table 1.

### 2.1. Population estimates

Population estimates for all risk groups were publicly available except for persons with chronic illnesses (excluding HIV) and pregnant women [31,32]. For pregnant women we used the total live births from mid-year 2016 population estimates [31]. To estimate the number of HIV-uninfected children and adults aged 5–64 years with chronic illness we obtained data on self-reported chronic illness from the South Africa National Health and Nutrition Survey (SA NHANES) [33]. The SA NHANES survey did not collect data on HIV status. To adjust for the increased prevalence of chronic conditions among HIV-infected children and adults we used the following formula [34]: the prevalence of underlying conditions in HIV-uninfected children and adults by age group = SA NHANES survey age group-specific prevalence of underlying conditions/[1 + 0.58 (age group-specific HIV-prevalence)]. We applied this prevalence estimate by age group (5–14y, 15–24y, 25–44y, and 45–64y) to the total HIV-uninfected population in the age stratum to estimate the population who would potentially seek care in non-HIV chronic illness clinics.

### 2.2. Rates of influenza-associated hospitalization

Rates of influenza-associated hospitalization were calculated using hospital-based surveillance at sentinel sites adjusting for non-enrolment, health-seeking behavior, and the attributable fraction of influenza-associated disease, stratified by age and HIV status [35,36]; see also Supplemental information). Rates were adjusted for specific risk factors (e.g. pregnancy, chronic illness) based upon the increased odds of influenza-associated hospitalization derived from hospital-based surveillance and data on the age-adjusted prevalence of the risk factor in the general population, or from the literature [11,37,38].

### 2.3. Rates of influenza-associated death

Influenza-associated deaths were estimated from published ecological models of seasonal excess mortality from vital statistics data using laboratory-confirmed influenza as a covariate [16,19,39,40]. Monte Carlo simulations using 5000 iterations based on a Poisson distribution in SAS 9.3 (SAS Institute, Cary, NC) were used to develop means and 95% confidence intervals for estimates of hospitalization or mortality rates requiring adjustment for risk factors.

### 2.4. Vaccine efficacy estimates

Vaccine efficacy (VE) of inactivated influenza vaccine was obtained from meta-analyses of the published literature for healthy adults and the elderly [23,41]. For pregnant women [15,25], infants aged 0–5 months [3,15,25], and children aged 6–23 months [22,42] we performed random effects meta-analyses of published randomized controlled trials (RCTs) of influenza VE (see results in Supplemental Information). There were no estimates of influenza VE in persons with tuberculosis disease and there are few RCTs that evaluate VE against laboratory-confirmed influenza infection in persons with chronic illnesses as most have looked at a single disease and at alternative endpoints (exacerbations, etc.). For this reason, we used the VE estimate for adults aged 65 years and older to score these two risk groups (Table 2). We assumed VE against influenza-associated hospitalization or death was equal to VE against laboratory-confirmed influenza-associated illness.

### 2.5. Vaccine coverage estimates

Each year the South African National Department of Health produces estimates of influenza vaccine coverage by target group. Target groups with estimated vaccine coverage in 2015 were pregnant women, children age 6–59 months, adults aged ≥65 years and persons with chronic illness (including HIV-infected adults) [43]. We did not find a local estimate of influenza vaccine coverage among healthcare workers so we used an average estimate of 10% from low and middle-income countries from the literature [44].

### 2.6. Coverage-adjusted hospitalization and mortality scores by risk group

To define target vaccine coverage we modified estimates of vaccine coverage from other vaccines targeting similar risk groups (e.g. tetanus vaccine coverage for pregnant women) [45] assuming an annual campaign of approximately 3 months duration^2^ would cover 50% of the annual coverage or, for groups with no current immunization platform, 80% of individuals presenting to a clinic in a 3-month period (20% for persons with HIV, TB and other chronic illness, 10% for persons aged ≥65 years). We assumed that healthcare worker vaccination coverage could be increased to 80% with improved access and education [44].

### 2.7. Estimated rate of hospitalizations/deaths averted by vaccination

We used the following formula to estimate the rate of hospitalizations (or deaths) averted by vaccination in each risk group: rate of influenza-associated hospitalization (or death) per 100,000 population * influenza vaccine efficacy. Monte Carlo simulations using 5000 iterations based on a Poisson distribution in SAS 9.3 (SAS Institute, Cary, NC) were used to develop means and 95% confidence intervals for estimates of the rate of influenza-associated hospitalizations (or deaths) averted per 100,000 vaccinated and an overall population-weighted estimate of the rate of influenza-associated hospitalizations (or deaths) averted per 100,000 vaccinated. The parameters used for the calculations are provided in Table 2.

### 2.8. Cost per hospital day averted/year of life saved

We also examine the potential years of life saved by vaccinating 100,000 individuals in each target group and the cost per year of life saved. The cost of vaccinating 100,000 individuals was estimated as the single dose vaccine price (~USD3) * 100,000 for all groups except children aged 6–23 months for whom the price was doubled^3^ [46]. Mean duration of hospitalization was estimated from local severe respiratory illness surveillance data. The cost per hospital day averted was calculated by dividing the cost of vaccinating 100,000 individuals by the product of the mean hospitalization duration and the estimated hospitalizations averted per 100,000 vaccinated. Estimated years of life saved was calculated for each risk group based upon expectation of life in years by age [47] and HIV-status [48]. The cost per year of life saved was calculated by dividing the cost of vaccinating 100,000 individuals by the product of the mean years of life saved per death averted and the estimated deaths averted per 100,000 vaccinated. The estimated hospitalizations and deaths averted by the current program and the target influenza vaccination programs were determined by multiplying the number of doses administered/100,000 by the rate of hospitalizations and deaths averted per 100,000 vaccinated.

## 3. Results

### 3.1. Hospitalizations averted per 100,000 vaccinated

Pregnant women had the highest estimated rate of hospitalizations averted per 100,000 vaccinated at 228.5 (95% CI 130.8–362.4) in addition to the benefit conferred to their infants aged 0–5 months (Table 2). Adults with HIV-infection, adults and children with tuberculosis disease, and adults aged 65 years or older also had high rates of hospitalization averted per 100,000 vaccinated at 190.9 (95% CI 24.5–390.6), 162.0 (95% CI 96.6–245.1), and 111.0 (95% CI 70.3–160.7), respectively. Young children aged 6–23 months had high rates of hospitalization averted per 100,000 vaccinated with wide confidence intervals (111.6, 95% CI 1.1–238.3) due to variable vaccine efficacy. Healthcare workers and adults and children aged 5–64 years with chronic illness had lower rates of hospitalization averted per 100,000 vaccinated at 36.1 (95% CI 26.5–46.3) and 29.4 (95% CI 15.5–44.7), respectively.

### 3.2. Deaths averted per 100,000 vaccinated

In South Africa, the majority of influenza-associated deaths occur in older adults and this resulted in adults aged ≥ 65 years having the highest estimated rate of deaths averted per 100,000 vaccinated, 96.6 (95% CI 26.2–180.9). Adults and children with TB disease also had very high rates of deaths averted per 100,000 vaccinated, 95.0 (95% CI 64.0–127.3). HIV-infected adults also had moderately high rates of deaths averted per 100,000 vaccinated, 18.2 (95% CI 6.1–102.3). Due to lower mortality rates, pregnant women, young children, healthcare workers and those with non-HIV chronic illness had lower estimates of deaths averted per 100,000 (Table 2).

### 3.3. Coverage-adjusted hospitalization and mortality scores by risk group

Many LMIC, including South Africa, provide immunization services to young children and pregnant women but few may offer immunization services to other risk groups. Therefore, countries introducing influenza vaccination programs may have to assess the opportunity costs associated with vaccinating persons outside these existing programs. We adjusted hospitalization and mortality rates per 100,000 by current and proposed target vaccine coverage to include an assessment of feasibility in our discussion of prioritization. Based upon coverage-adjusted hospitalization and mortality scores pregnant women remain the highest priority for averting hospitalizations (target score 102.5); adults and children with TB disease and healthcare workers increase in relative prioritization based upon target mortality scores (scores 19.0 and 10.0, respectively) (Table 3). HIV-infected adults remain an important risk group in both coverage-adjusted hospitalization and mortality scores, and the elderly remain an important risk group based upon mortality scores.

### 3.4. Cost per hospital day averted and cost per year of life saved

The cost per hospital day averted ranged from USD148–1,344 with a population-weighted mean of USD386 (95% CI 244–761) (Table 4). Cost per hospital day averted was lowest among pregnant women and their infants (USD148), HIV-infected adults (USD196), and adults and children with TB (USD206). The highest costs per hospital day averted were in children aged 6–23 months (USD1,344) and adults and children aged 5–64 years with non-HIV, non-TB chronic illnesses (USD1,276). The cost per year of life saved ranged from USD112–1,230 with a population weighted mean of USD429 (95% CI 226–1,425) and was lowest among adults and children with TB (USD112), HIV-infected adults (USD257), and pregnant women (USD373) (Table 4).

### 3.5. Estimated current and target vaccine coverage and impact

The estimated number of South Africans that fall into one or more risk categories was over 23 million in 2016. Current vaccination coverage in the public sector is less than 5% in all risk groups except pregnant women (14%). Despite relatively low coverage, our simple model estimated 1054 (95% CI 405–1,849) hospitalizations and 247 (95% CI 99–431) deaths might have been averted in 2016. At approximately USD3 per dose of influenza vaccine, the cost per hospitalization averted was approximately USD2,386 (95% CI 1,360–6,203) and the cost per death averted was USD10,200 (95% CI 5,836–25,426). If resources allocated to influenza vaccine procurement were increased, the proposed target program could potentially avert 5,538 (95% CI 1,722–10,126) hospitalizations and 1,478 (95% CI 545–2,656) deaths (Table 5). At the same cost per dose, the cost per hospitalization averted would be approximately USD2,781 (95% CI 1,521–8,943) and the cost per death averted would be USD10,420 (95% CI 5,800–28,270).

## 4. Discussion

Hospitalization rates and mortality rates due to influenza-associated illness may differ in LMIC, there are cultural differences in care-seeking for severe respiratory illness, and there are few public sector programs that routinely provide immunization and preventive care services. Considering all of these factors in prioritization of risk groups for influenza vaccine introduction is essential to program acceptance and success in resource-limited settings. Prior studies of influenza vaccine prioritization have focused on minimizing years of life lost [49], on individuals at high-risk of complications [50] or on reducing transmission by vaccinating children [51] during pandemics. This analysis is a first attempt at applying similar concepts to routine annual influenza vaccination.

In South Africa, adults and children with TB, HIV-infected adults, and pregnant women had among the lowest costs per hospital day averted and year of life saved. While the elderly rank highest when considering potential deaths averted per 100,000 vaccinated, their lower hospitalization rates and lower years of life lost per death increased the cost per hospital day averted and per year of life saved. Lower vaccine efficacy and the need for 2 doses of influenza vaccine in children aged 6–23 months who have not been previously vaccinated increase the cost per hospital day averted and per year of life saved for this risk group.

There are several limitations to this methodology. First, data on vaccine efficacy in some risk groups were extremely limited. There were no data on influenza vaccine efficacy in persons with tuberculosis disease. Vaccine efficacy data in children aged 6–23 months and HIV-infected adults were also limited. Second, we assumed influenza vaccine efficacy would be the same for influenza-associated hospitalization or death as it is against laboratory-confirmed influenza-associated illness, where most data are collected among outpatients. Third, our estimates of cost per hospital day averted or year of life saved do not include adjustments for vaccine delivery or administration. It was challenging to separate administration costs because there were no additional staff hired to administer immunizations during annual influenza vaccination campaigns. Fortunately, administration and transportation costs should be similar across risk groups and would therefore be less likely to affect relative prioritization. Fourth, we only assessed the potential impact of trivalent inactivated influenza vaccines. Newer vaccines that may differentially increase vaccine efficacy in risk groups might impact the relative prioritization of risk groups. Finally, the results are based on prevention of influenza-associated hospitalization or death and do not assess potential impact of vaccination on disease transmission or productivity loss. Including indirect effects of vaccination on influenza transmission might improve overall estimates of cost effectiveness or result in different prioritization of risk groups.

Prior modelling of the impact of influenza vaccination on disease burden in pregnant women and infants was able to inform estimates of the fraction of infant disease potentially averted through maternal vaccination [52] (M Biggerstaff, personal communication). For other risk groups we assumed that vaccine effectiveness was equivalent to published estimates of efficacy. This assumption may be incorrect in that not all persons may have received vaccination prior to the onset of the influenza season, vaccine effectiveness varies depending on how well the vaccine strains match the circulating strains, and vaccine effectiveness may wane during the season.

South Africa has published rates of influenza-associated illness and death in most of the vaccine target groups. Likewise, population estimates from census data, disease modelling, or other sources were available for most risk groups. However, there were few published estimates of the prevalence of common co-morbid conditions in South Africa and none that assessed the prevalence of any co-morbid condition (i.e. one or more conditions). By using the SA NHANES survey data we estimated the prevalence of any common chronic illness; however, the SA NHANES did not document all underlying illnesses nor the HIV status of participants. We attempted to adjust for the increased prevalence of comorbid conditions among HIV-infected children and adults in estimating the prevalence of other chronic illnesses in HIV-uninfected children and adults but there were no data available on such adjustments in a LMIC setting.

Finally, we did not include formal cost-effectiveness analyses for each risk group. We have recently collected costing data for respiratory illnesses in South Africa and hope to produce estimates of influenza vaccine cost effectiveness in the future.

## 5. Conclusions

In 2016, the National Advisory Group on Immunization in South Africa recommended that pregnant women and HIV-infected persons be prioritized for publicly funded influenza vaccination given available evidence on influenza-associated disease burden and a preliminary version of this analysis (S. Walaza, personal communication). These two risk groups had the highest rates of potential hospitalizations averted and were among the lowest cost per hospital day averted and year of life saved. Only adults and children with TB disease had lower cost per hospital day averted and year of life saved but this group was not prioritized given the lack of available data on influenza vaccine efficacy.

## Supplementary Material

2

## Figures and Tables

**Table 1:** Sources of data on priority risk groups for influenza vaccination in South Africa, 2016.

Data	Source	Estimation method
*Population Estimates*		
Population estimate for pregnant women	Statistics South Africa Mid-Year Population Estimates, 2016 [31]	Assumed equal to annual live births
Population estimate for HIV-infected adults aged 15–64 years	Statistics South Africa Mid-Year Population Estimates, 2016 [31]; Thembisa Model [32]	Applied 2016 HIV prevalence by year of age from the Thembisa model to mid-year population estimates for individuals aged15–64 years
Population estimate for children aged 6–23 months	Statistics South Africa Mid-Year Population Estimates, 2016 [31]	Sum of half of infants aged 0–11 months plus all children aged 12–23 months
Population estimate for adults aged ≥ 65 years	Statistics South Africa Mid-Year Population Estimates, 2016 [31]	Sum of adults aged ≥ 65 years
Population estimate for healthcare workers	Health Professions Council of South Africa (HPCSA) [53]; South African Nursing Council (SANC) [54]; Shisana et al. [55]	Sum of registered providers from HPCSA (3 May 2016) and nurses from SANC (2015); HIV prevalence in healthcare workers
Population estimate for adults and children with TB disease	WHO World TB Report 2015, South Africa country profile [56]	Assumed no change from 2014 estimate
Population estimate for adults and children aged 5–64 years with other chronic disease	South Africa National Health and Nutrition Survey (SA NHANES) [33]; Schouten et al. [34]	Determined age-standardized prevalence of 1 or more of the following underlying conditions: Adults aged 15–64 years – cardiovascular disease, diabetes, history of tuberculosis, more than 10 pack-years smoking, or BMI ≥ 35; Children aged 0–14 years – BMI < 97%ile, history of >3% weight for age, or seizure disorder. Adjusted for increased prevalence of comorbid conditions in HIV-infected compared to HIV-uninfected, odds ratio 1.58 (95% CI 1.23–2.03).
*Hospitalization Rates*		
Influenza-associated hospitalization rate in pregnant women	Tempia et al. [35]; Van Kerkhoven et al. [11]	Age and HIV-standardized rates were used to estimate influenza-associated hospitalization rates for adults aged 15–49 years and adjusted for the increased risk of influenza-associated hospitalization among pregnant women: relative risk 6.8 (95% CI 4.5–12.3).
Influenza-associated hospitalization rate in infants aged 0–5 months	McMorrow et al. [36]	Rate from local data for children aged 0–5 months, 2011–2016
Influenza-associated hospitalization rate in HIV-infected adults aged 15–64 years	Tempia et al. [35]	Rate from local data for HIV-infected adults aged 15–64 years, 2013– 2015
Influenza-associated hospitalization rate in children aged 6–23 months	McMorrow et al. [36]	Unpublished rate from local data for children aged 6–23 months, 2011–2016
Influenza-associated hospitalization rate in adults aged ≥ 65 years	Tempia et al. [35]	Rate from local data for adults aged ≥ 65 years, 2013–2015
Influenza-associated hospitalization rate in healthcare workers	Tempia et al. [35]; SANC [54]; Shisana et al. [55]	Unpublished rate from local data, 2013–2015 adjusted for age distribution and HIV-prevalence of healthcare workers
Influenza-associated hospitalization rate in adults and children with TB disease	Lawn et al. [57]; WHO World TB Report 2015, South Africa country profile [56]; Tempia et al. [35]; Abadom et al. [37]	Age-adjusted TB incidence was estimated from the literature. HIV prevalence was assumed to be 60% among TB-infected adults and children. Age and HIV-standardized rates were adjusted for the increased odds of influenza-associated hospitalization from recently published local data: case-population ratio 1.85 (95% CI 1.68–2.02).
Influenza-associated hospitalization rate in adults and children aged 5–64 years with other chronic disease	Tempia et al. [35]; Abadom et al. [37]	Age and HIV-standardized rates were adjusted for the increased odds of influenza-associated hospitalization in those with chronic illness (assumed from hospitalization in last year) compared to HIV-uninfected adults from local data: case-population ratio 2.07 (95% CI 1.92–2.23).
*Death rates*		
Influenza-associated death rate in pregnant women	Tempia et al. [39]	Published rate from local data, 1999–2009
Influenza-associated death rate in infants aged 0–5 months	Cohen et al. [40]	Unpublished rate from local data for infants aged 0–11 months, 2009–2013
Influenza-associated death rate in HIV-infected adults aged 15–64 years	Tempia et al. [16]	Published rate from local data, 1998–2009
Influenza-associated death rate in children aged 6–23 months	Cohen et al. [40]	Unpublished rate from local data for children aged 0–11 months and 1–4 years, 2009–2013
Influenza-associated death rate in adults aged ≥ 65 years	Cohen et al. [40]	Unpublished rate from local data, 2009–2013
Influenza-associated death rate in healthcare workers	Cohen et al. [40]	Unpublished rate from local data for adults aged 20–64 years, 2009–2013
Influenza-associated death rate in adults and children with TB disease	Walaza et al. [19]	Published rate from local data, 1999–2009
Influenza-associated death rate in adults and children aged 5–64 years with other chronic disease	Cohen et al. [40]; Mertz et al. [38]	Unpublished rate from local data for children and adults aged 5–64 years, 2009–2013 adjusted for increased odds of death among persons with chronic illness: odds ratio 2.04 (95% CI 1.74–2.39).
*Vaccine efficacy*		
Vaccine efficacy estimate for pregnant women	Madhi et al. [15] Tapia et al. [25]	Estimated using 1-risk ratio (RR). RR estimated using random effects Mantel-Haenszel model of published randomized controlled trials of inactivated influenza vaccine in pregnant women.
Maternal influenza vaccine efficacy against influenza-associated illness in infants aged 0–5 months	Madhi et al. [15] Tapia et al. [25] Zaman et al. [3]	Estimated using 1-risk ratio (RR). RR estimated using random effects Mantel-Haenszel model of published randomized controlled trials of inactivated influenza vaccine in pregnant women.
Vaccine efficacy estimate for HIV-infected adults	Madhi et al. [58]	Assumed no change.
Vaccine efficacy estimate for children aged 6–23 months	Hoberman et al. [42]Vesikari et al. [22]	Estimated using 1-RR. RR estimated using random effects Mantel-Haenszel model of published randomized controlled trials of inactivated influenza vaccine in children aged 6–23 months.
Vaccine efficacy estimate for adults aged ≥ 65 years	Darvishian et al. [23]	Meta-analysis of 35 test-negative design case-control studies
Vaccine efficacy estimate for healthcare workers	Demicheli et al. [41]	Assumed equivalent to healthy adults in Cochrane Review and meta-analysis of 20 RCTs
Vaccine efficacy estimate for adults and children with TB	Darvishian et al. [23]	No published studies of VE in persons with TB; we used the VE estimate from adults aged 65 years and older.
Vaccine efficacy estimate for adults and children aged 5–64 years with chronic illnesses	Darvishian et al. [23]	Due to the limited data on vaccine efficacy among persons with chronic illnesses (Michiels et al. [59]) we used the VE estimate from adults aged 65 years and older.
*Vaccine coverage*		
Current influenza vaccine coverage estimates for all risk groups	Ramkrishna [43]	Assumed no change from 2015. HIV and TB-infected adults included in chronic disease estimates per author.
Current influenza vaccine coverage estimates for healthcare workers	Haviari et al. [44]	No estimate available for South Africa. Most low and middle income countries had vaccine low influenza vaccine coverage in this review so we chose 10% as an estimate of current influenza vaccine coverage in South African healthcare workers.
Current vaccine coverage estimates for measles second dose (children) and tetanus toxoid (pregnant women)	WHO/UNICEF South Africa estimates [45]	Assumed no change from 2015 estimate
*Other parameters*		
Proportion of HIV-infected currently in care and treatment	Thembisa model 2.5 [32]	Used model assumptions for 2016
Proportion of persons with TB disease currently receiving treatment	WHO World TB Report 2015, South Africa country profile [56]	Assumed no change from 2014 estimate
Cost of a single dose of inactivated influenza vaccine	Pan American Health Organization Revolving Fund Vaccine Prices for 2016 [46]	USD3 (range USD2.80–3.95)
Mean duration of influenza-associated hospitalization in days for each risk group	NICD unpublished data	Severe respiratory illness surveillance 2009–2016
Expectation of life in years by age	WHO Global Health Observatory [47]; Johnson et al. [48]	Assumed no change from 2013 estimates

**Table 2:** Summary of population size, influenza-associated hospitalization rates, influenza-associated death rates, vaccine efficacy, potential hospitalizations averted per 100,000 vaccinated, potential deaths averted per 100,000 vaccinated, and vaccine coverage by high risk group, South Africa.

Risk group	Mid-year population in South Africa (2016)	Age and HIV-standardized rate of influenza-associated hospitalization per 100,000 person years (95% CI)	Age and HIV-standardized rate of influenza-associated death per 100,000 person years (95% CI)	Vaccine efficacy* in risk group	Potential hospitalizations averted per 100,000 vaccinated (95% CI)	Potential deaths averted per 100,000 vaccinated (95% CI)	Vaccine coverage**
Pregnant women	1,198,861 (births) 270,235 HIV-infected (22.5%)	378.8 (237.7–534.8)	12.6 (7.2–18.0)	61% (95% CI, 43–73%) n = 6430	228.5 (130.8–362.4) 27.8 (15.3–40.6)***	7. 7(3.9–12.0) 7.8 (5.1–10.7)***	14% Tetanus toxoid 2 dose coverage 80% in 2015
HIV-infected adults aged 15–64 years	6,604,709	256.3 (179.4–333.2)	64.7 (38.3–81.1)	76% (95% CI, 9–96%), n = 506	190.9 (24.5–390.6)	48.2 (6.1–102.3)	3%
Children aged 6–23 months	1,729,502 15,911 HIV-infected (0.9%)	324.1 (237.6 – 417.8)	23.4 (5.1 – 26.5)	35% (95% CI, −41–70%), n = 1893	111.6 (1.1 – 238.3)	8.0 (0.1–18.3)	3% Measles vaccine 2 dose coverage 63% in 2015
Adults aged ≥ 65 years	2,909,122 97,640 HIV-infected (3.36%)	194 (144–256)	169.3 (81.0–324.6)	58% (95% CI, 40–70%), n = 11,848	111.0 (70.3 – 160.7)	96.6 (26.2–180.9)	2%
Healthcare workers	566,393 88,924 HIV-infected (15.7%)	60.6 (45.5 – 75.8)	20.9 (9.4–29.0)	60% (95% CI, 53–66%), n = 51,724	36.1 (26.5–46.3)	12.5 (6.6–18.7)	10%
Adults and children with TB (all ages)	318,193 notified cases in 2014 ( ≥ 60% HIV-infected)	282.7 (193.5–377.4)	164 (144–174)	Unknown; used estimate in elderly 58% (95% CI, 40–70%), n = 11,848	162.0 (96.6 – 245.1)	95.0 (64.0 – 127.3)	3%
Adults and children aged 5–64 years with other chronic illnesses	9,863,353	42.4 (31.8 – 53.7)	24.4 (15.4 – 35.3)	Unknown; used estimate in elderly 58% (95% CI, 40–70%), n = 11,848	29.4 (15.5 – 44.7)	16.7 (8.3–28.3)	3%

* Against laboratory-confirmed influenza-associated illness.

** 2015 national estimates for all risk groups except healthcare workers.

*** Due to timing of maternal vaccination only 8–10% of burden in infants may be averted (M. Biggerstaff, in preparation).

**Table 3:** Summary of 2016 vaccine coverage, target vaccine coverage and coverage-adjusted hospitalization and mortality scores by high risk group, South Africa.

Risk group	2015 vaccine coverage**	Current coverage-adjusted hospitalization score	Current coverage-adjusted mortality score	Target vaccine coverage	Target coverage-adjusted hospitalization score	Target coverage-adjusted mortality score
Pregnant women	14%	35.8 (20.5–56.4)	2.2 (1.3–3.2)	40%	102.5 (58.4–161.2)	6.2 (3.6–9.1)
HIV-infected adults aged 15–64 years	3%	5.7 (0.7–11.7)	1.4 (0.2–3.1)	20%	38.2 (4.9–78.1)	9.6 (1.2–20.5)
Children aged 6–23 months	3%	3.3 (0.0–7.1)	0.2 (0.0–0.5)	32%	35.6 (0.4–76.3)	2.9 (0.0–5.9)
Adults aged ≥ 65 years	2%	2.2 (1.4–3.2)	1.9 (0.5–3.6)	10%	11.1 (7.0–16.1)	9.7 (2.6–18.1)
Healthcare workers	10%	3.6 (2.6–4.6)	1.3 (0.7–1.9)	80%	28.8 (21.2–37.0)	10.0 (5.3–15.0)
Adults and children with TB (all ages)	3%	4.9 (2.9–7.4)	2.9 (1.9–3.8)	20%	32.4 (19.3–49.0)	19.0 (12.8–25.5)
Adults and children aged 5–64 years with other chronic illnesses	3%	0.9 (0.5–1.3)	0.5 (0.2–0.8)	20%	5.9 (3.1–8.9)	3.3 (1.7–5.7)

** 2015 national estimates for all risk groups except healthcare workers.

**Table 4:** Estimated cost per hospital day averted and cost per year of life saved by influenza vaccination in selected risk groups, South Africa, 2016.

Risk group	Mean duration of hospitalization in days (standard deviation)	Cost per hospital day averted (USD) (95% CI)	Average years of life lost per death	Average years of life saved per 100,000 vaccinated (95% CI)	Cost per year of life saved (USD) (95% CI)
Pregnant women -and- Infants aged 0–5 months	7 (9) 9 (9)	148 (103–285)	43 60	805 (477–1166)	373 (257–629)
HIV-infected adults aged 15–64 years	8 (9)	196 (96–1,531)	24	1166 (148–2476)	257 (121–2,032)
Children aged 6–23 months	4 (6)	1,344 (629–136,364)	61	488 (6–1116)	1230 (537–98,361)
Adults aged ≥ 65 years	8 (9)	338 (233–533)	7	628 (170–1176)	478 (255–1,762)
Healthcare workers	8 (8)	1,039 (810–1,415)	30	370 (195–554)	811 (542–1,536)
Adults and children with TB	9 (10)	206 (136–345)	28	2689 (1811–3603)	112 (83–166)
Adults and children aged 5–64 years with other chronic diseases	8 (9)	1,276 (839–2,419)	31	511 (254–866)	587 (346–1,181)
Total	8 (9)	386 (244–761)	32	752 (226–1427)	429 (226–1,425)

**Table 5:** Estimated hospitalizations and deaths potentially averted in current and target influenza vaccination programs, South Africa, 2016.

Risk group	Number vaccinated by current program (% target population)	Estimated hospitalizations averted by current program (95% CI)	Estimated deaths averted by current program (95% CI)	Number vaccinated with target coverage* (% target population)	Estimated hospitalizations averted by target program (95% CI)	Estimated deaths averted by target program (95% CI)
Pregnant women -and- Infants aged 0–5 months	167,840 (14)	430 (245–676)	26 (15–38)	479,544 (40)	1229 (701–1933)	74 (43–109)
HIV-infected adults aged 15– 64 years	198,141 (3)	378 (49–774)	96 (12–203)	1,320,942(20)	2522 (324–5160)	637 (81–1351)
Children aged 6–23 months	51,885 (3)	58 (1–124)	4 (0–9)	553,441 (32)	618 (6–1319)	44 (1–101)
Adults aged ≥ 65 years	58,182 (2)	65 (41–93)	55 (37–74)	290,912 (10)	323 (205–467)	276 (186–370)
Healthcare workers	56,639 (10)	20 (15–26)	7 (4–11)	453,114 (80)	164 (120–210)	57 (30–85)
Adults and children with TB	9,546 (3)	15 (9–23)	9 (6–12)	63,639 (20)	103 (61–156)	60 (41–81)
Adults and children with other chronic illnesses	295,901 (3)	87 (46–132)	49 (25–84)	1,972,671 (20)	580 (306–882)	329 (164–558)
Total	838,134 (4)	1054 (405–1849)	247 (99–431)	5,134,263 (22)	5,538 (1,722–10,126)	1,478 (545–2,656)

* Target vaccine coverage derived from 50% coverage with an existing vaccination platform or assumption of 80% coverage of individuals presenting over a 3-month vaccination period.

## Footnotes

1 In this analysis, we included children aged 6–23 months rather than children aged 6–59 months because few children aged 24–59 months are routinely seen in vaccination clinics in South Africa.

2 Unpublished data from the South African Department of Health demonstrate that during 2015–2017 75–96% of influenza vaccines administered were delivered within 3 months, typically April-June.

3 Cost was doubled for children aged 6–23 months because they require 2 doses of influenza vaccine.
