# Supplementary material for: Prioritization of risk groups for influenza vaccination in resource limited settings – A case study from South Africa
Source: Vaccine. Author manuscript; Available in PMC 2019 Jul 3. (PMC6470296; doi:10.1016/j.vaccine.2018.11.048)
Supplement: 2 [file NIHMS1012564-supplement-2.docx]

Supplemental information for “**Prioritization of risk groups for influenza vaccination in resource limited settings – a case study from South Africa”**

Methods:

Vaccine efficacy

Where vaccine efficacy data were published for risk groups but no meta-analyses were available, vaccine efficacy was estimated using 1-risk ratio (RR). The RR was estimated by performing a random effects meta-analysis using the metan command in STATA version 14.1 (StataCorp, College Station, Texas, USA). Results of these meta-analyses are presented below.

Pregnant women:

Infants aged 0-5 months:

Children aged 6-23 months:

Hospitalization rates:

All rates are expressed per 100,000 population

**Pregnant women (15-49 years of age):**

378.8 (237.7-534.8)

For this calculation I used the following procedure:

1. Obtain total number of SRI (from logs), number of SRI tested and number of flu+ (from SARI database) among adults aged 15-49 years (within the relevant age categories).
2. Estimate influenza-associated rates as described in Tempia et al. (1000 bootstrap iterations using Stata version 14.1) among HIV+/HIV- individuals separately.
3. Apply the HIV prevalence among pregnant women to the source population of adults aged 15-49 years.
4. Apply rates among HIV+ and HIV- adults aged to 15-49 years (obtained in step 2) to the source population reflecting the HIV prevalence among pregnant women to obtain the number of cases in each HIV stratum.
5. Sum the number of estimated HIV+ and HIV- cases.
6. Divide the total number of cases (irrespective of HIV status) by the population at risk to obtain rates.
7. Adjust rates by relative risk of influenza-associated hospitalization among pregnant women 6.8 (95% CI 4.5-12.3) with Monte Carlo simulations using 5000 iterations based on a Poisson distribution in SAS 9.3 (SAS Institute, Cary, NC) to develop means and 95% confidence intervals.
8. The 95% CIs are the 2.5^th^ and 97.5^th^ percentiles of the bootstrapped datasets.

**HIV+ adults (15-64 years of age)**

- 256.3 (95% CI: 179.4-333.2)

For this calculation I used the following procedure:

1. Obtain total number of SRI (from logs), number of SRI tested and number of flu+ (from SARI database) among adults aged 15-64 years (within the relevant age categories).
2. Estimate influenza-associated rates as described in Tempia et al. (1000 bootstrap iterations) among HIV+/HIV- individuals separately in this age group.
3. The 95% CIs are the 2.5th and 97.5th percentiles of the 1000 bootstrapped datasets.

**Children aged 6-23 months:**

- 324.1 (237.6 – 417.8)

1. Obtain total number of SRI (from logs), number of SRI tested and number of flu+ (from SARI database) among children aged 6-23 months (within the relevant age categories).
2. Estimate influenza-associated rates as described in Tempia et al. (1000 bootstrap iterations using Stata version 14.1) among HIV+/HIV- individuals separately.
3. Apply the HIV prevalence among children aged 0-11 and 12-23 months of age to each group.
4. Apply rates among HIV+ and HIV- children aged 6-11 and 12-23 months (obtained in step 2) to the source population reflecting the HIV prevalence among infants aged 0-11 months and children aged 12-23 months to obtain the number of cases in each HIV stratum.
5. Sum the number of estimated HIV+ and HIV- cases.
6. Divide the total number of cases (irrespective of HIV status) by the population at risk to obtain rates.
7. The 95% CIs are the 2.5th and 97.5th percentiles of the 1000 bootstrapped datasets.

**Adults aged ≥65 years:**

- 194 (144 - 256)

As reported in Tempia et al.

**HCW (15-64 years of age):**

- 60.6 (95% CI: 45.5-75.8)

For this calculation I used the following procedure:

1. Obtain total number of SRI (from logs), number of SRI tested and number of flu+ (from SARI database) among adults aged 15-64 years.
2. Estimate influenza-associated rates as described in Tempia et al. (1000 bootstrap iterations) among HIV+/HIV- individuals separately.
3. Apply the HIV prevalence among HCW to the source population of adults aged 15-64 years.
4. Apply rates among HIV+ and HIV- adults aged to 15-64 years (obtained in step 2) to the source population reflecting the HIV prevalence among HCW to obtain the number of cases in each HIV stratum.
5. Sum the number of estimated HIV+ and HIV- cases.
6. Divide the total number of cases (irrespective of HIV status) by the population at risk to obtain rates.
7. The 95% CIs are the 2.5^th^ and 97.5^th^ percentiles of the 1000 bootstrapped datasets.

**TB (all ages):**

282.7 (193.5-377.4)

For this calculation I used the following procedure:

1. Use influenza-associated rates for individuals aged <5, 5-24, 24-44, 45-64 and 65+ years reported in my paper (1000 bootstrap iterations) among HIV+/HIV- individuals separately.
2. Apply rates among HIV+ and HIV- individuals to the TB population reflecting an HIV prevalence of 60% among all age groups to obtain the number of cases in each HIV stratum.
3. Sum the number of estimated HIV+ and HIV- cases.
4. Divide the total number of cases (irrespective of HIV status) by the TB population to obtain rates.
5. Adjust this rate by the increased odds of influenza-associated hospitalization in persons with TB from Abadom et al. case-population ratio 1.85 (95% CI 1.68-2.02) with Monte Carlo simulations using 5000 iterations based on a Poisson distribution in SAS 9.3 (SAS Institute, Cary, NC) to develop means and 95% confidence intervals.
6. The 95% CIs are the 2.5^th^ and 97.5^th^ percentiles of the bootstrapped datasets.

**HIV- Individuals 5-64 years of age with chronic illness:**

- 42.4 (31.8 – 53.7)

1. For this calculation I just calculated an aggregated rate as the extremes of the age group are the same as those in Tempia et al.
2. Adjust this rate by the increased odds of influenza-associated hospitalization in persons with chronic illness from Abadom et al. case-population ratio 2.07 (95% CI 1.92-2.23) with Monte Carlo simulations using 5000 iterations based on a Poisson distribution in SAS 9.3 (SAS Institute, Cary, NC) to develop means and 95% confidence intervals.
3. The 95% CIs are the 2.5^th^ and 97.5^th^ percentiles of the bootstrapped datasets.
